# Smartphone use is a risk factor for pediatric dry eye disease according to region and age: a case control study

**DOI:** 10.1186/s12886-016-0364-4

**Published:** 2016-10-28

**Authors:** Jun Hyung Moon, Kyoung Woo Kim, Nam Ju Moon

**Affiliations:** Department of Ophthalmology, College of Medicine, Chung Ang University Hospital, 102, Heukseok-ro, Dongjak-gu, Seoul, 06973 Korea

**Keywords:** Dry eye disease, Pediatrics, Smartphone, Outdoor activity, Video display terminal

## Abstract

**Background:**

In 2014, the overall rate of smartphone use in Korea was 83 and 89.8 % in children and adolescents. The rate of smartphone use differs according to region (urban vs. rural) and age (younger grade vs. older grade). We investigated risk and protective factors associated with pediatric dry eye disease (DED) in relation to smartphone use rate according to region and age.

**Methods:**

We enrolled 916 children and performed an ocular exam that included slit lamp exam and tear break-up time. A questionnaire administered to children and their families consisted of video display terminal (VDT) use, outdoor activity, learning, and modified ocular surface disease index (OSDI) score. DED was defined based on the International Dry Eye Workshop guidelines (Objective signs: punctate epithelial erosion or short tear break-up time; subjective symptoms: modified OSDI score) We performed statistical analysis of risk factors and protective factors in children divided into groups as follows: DED vs. control, urban vs. rural, younger grade (1st to 3rd) vs. older grade (4th to 6th).

**Results:**

A total of 6.6 % of children were included in the DED group, and 8.3 % of children in the urban group were diagnosed with DED compared to 2.8 % in the rural group (*P* = 0.03). The rate of smartphone use was 61.3 % in the urban group and 51.0 % in the rural group (*P* = 0.04). In total, 9.1 % of children in the older-grade group were diagnosed with DED compared to 4 % in the younger-grade group (*P* = 0.03). The rate of smartphone use was 65.1 % in older-grade children and 50.9 % in younger-grade children (*P* < 0.001). The mean daily duration of smartphone use was longer in the DED group than controls (logistic regression analysis, *P* < 0.001, OR = 13.07), and the mean daily duration of outdoor activities was shorter in the DED group than controls (logistic regression analysis, *P* < 0.01, OR = 0.33). After cessation of smartphone use for 4 weeks in the DED group, both subjective symptoms and objective signs had improved.

**Conclusions:**

Smartphone use in children was strongly associated with pediatric DED; however, outdoor activity appeared to be protective against pediatric DED. Older-grade students in urban environments had DED risk factors (long duration of smartphone use), and a short duration of outdoor activity time. Therefore, close observation and caution are needed when older children in urban areas use smartphones.

**Electronic supplementary material:**

The online version of this article (doi:10.1186/s12886-016-0364-4) contains supplementary material, which is available to authorized users.

## Background

Dry eye disease (DED) can occur in association with a number of congenital, autoimmune, endocrine, and inflammatory disorders, or under certain environmental and nutritional conditions [1]. We initiated this study to investigate environmental risk factors and protective factors for pediatric dry eye disease to correct risk factors and encourage protective factors.

Korea has one of the world’s highest smartphone use rates with an estimated 89.8 % rate of usage in 2014. Pediatric dry eye disease tends to have a higher prevalence in younger ages [1]. Therefore, it is imperative to investigate risk factors and protective factors for pediatric DED to prevent disease progression as much as possible.

Use of video display terminals and learning (reading and writing) for long hours has been associated with a decreased maximum blink interval, hence the development of dry eye symptoms [2–4]. In addition, many people report ocular fatigue after prolonged work with video display terminals, in part prompting legislation regulating the use of these terminals [5].

Pediatric DED is a public health concern and economic challenge; thus, it is important to dedicate considerable clinical attention to pediatric DED in Korea [6]. In our previous study, we investigated the association between pediatric DED and video display terminal use [7]. DED is a multi-factorial disease, and there have not yet been any in-depth studies considering risk factors and protective factors for pediatric DED according to age and region. In our study, we investigated risk factors and protective factors for pediatric dry eye disease associated with smartphone use rate according to region and age.

## Methods

### Subjects

This study was a school-based cross sectional, case-control study. We performed ophthalmologic examinations on 630 children from eight primary schools in Seoul, Korea (urban, male:female = 318:312) and in 286 children in Paju, Korea (rural, male:female = 168:118) from May through October 2015. Seoul is the capital city of South Korea and a large urban center with a population of approximately 10 million. Paju is a city with a population near 400,000. We performed ocular exams on children in younger (1st to 3rd grade, aged 7 to 9) and older (4th to 6th grade, aged 10 to 12) grades from elementary schools in these two regions.

This study was performed according to the Declaration of Helsinki on Biomedical Research Involving Human Subjects. The Institutional Review Board of Chung Ang University of Medicine Review Board approved the clinical study. Both parents and children themselves provided written informed consent after being given a detailed explanation of the study. We confirmed that parents had seen the manuscript and patient data and agreed to its publication in a journal.

The relative humidity in this region of Korea during the time of the study (May to October) ranged from 57.8 to 58.2 % in urban areas (Seoul) and 58.1 to 58.4 % in rural areas (Paju). There was no significant difference in relative humidity between the two regions.

Exclusion criteria were: (1) children who underwent any type of eye surgery in the past 6 months, (2) children who had nocturnal lagophthalmos, (3) children who had eyelid problems (trichiasis, districhiasis, or epiblepharon), (4) children who had allergic conjunctivitis with the use of antihistamine drugs, (5) children who wear contact lenses, and (6) children who had congenital, endocrinal, or autoimmune disease.

### Ocular examinations

A single examiner performed all ocular exams. These included visual acuity tests (best corrected VA with trial lens, LogMAR), autokeratomery, slit-lamp examinations of the cornea and conjunctiva, and evaluation of eyelid problems, allergic conjunctivitis, and exposure keratitis. Autokeratometry was measured using Topcon Auto Kerato-Refractometer KR-8800 by a single examiner; we reported the mean value of the three repeated measurements.

Tear break-up time (TBUT) was measured with a fluorescein strip (Haag-Streit Inter- national, Koniz, Switzerland) coated with one drop of balanced salt solution (BSS; Alcon Laboratories, Inc., Fort Worth, TX). After applying the strip to the inferior conjunctival fornix, the participant resumed normal blinking for several seconds. After the fluorescein solution spread across the corneal surface, the participant was asked to keep his or her eye open until the first defect of the tear film occurred. TBUT was defined as the interval between the last complete blink and the first appearance of a dry spot on the pre-corneal surface of the tear film [8]. The procedure was repeated three times for each eye tested, with results reported as the mean value of the three measurements.

A single examiner evaluated punctate epithelial erosions (PEE) in the cornea and conjunctiva using a slit lamp according to the Oxford Scheme Panel from 0 to 5 grades [9]. The study protocol is included (Additional file 1).

### Questionnaires

A self-administered questionnaire was given to the children and their parents and both children and their parents completed each questionnaire. Questionnaires were designed to obtain information regarding risk factors for dry eye disease, including the mean daily duration of video display terminal (smartphone, computer, television) use, learning (reading and writing), outdoor activities, and past history of allergic disease and antihistamine drug use. Subjective ocular symptoms were measured with the modified ocular surface disease index (OSDI) score. This index was an objective parameter for DED diagnosis. Modified OSDI scores range from 0 to 100 points, and higher scores indicate greater discomfort due to dry eye disease. We added the OSDI index and ophthalmologic questionnaire (Additional file 1).

### Diagnostic criteria of dry eye disease

Dry eye disease was diagnosed using a combination of questionnaire data and clinical ophthalmologic testing that elicited information on signs and symptoms based on the 2007 Dry Eye Workshop (DEWS) guidelines [10].

Objective signs included (1) TBUT less than 10 s or (2) positive corneal and conjunctival fluorescein staining. A modified OSDI score greater than 20 points was used for a positive measure of subjective symptoms. Children who had one or more objective signs and more than 20 points on the modified OSDI score were analyzed in the DED group.

### Statistical analysis

We divided the school children into two groups and made the following comparisons: DED vs. control, urban vs. rural, younger graders vs. older graders. We used multivariate logistic regression analysis to assess DED risk and protective factors. Then, during 4 weeks of smartphone cessation in the DED group, we compared before and after parameters. Statistical analyses were performed using SPSS for Windows (version 21; SPSS, Inc., Chicago, IL). Continuous variables were compared between groups using the Student’s t-test. The chi-square test was used to compare non-continuous variables between the two groups. *P* values less than 0.05 were considered statistically significant.

## Results

### Comparative analysis between control and dry eye disease group

The rate of DED in all children was 6.6 %. There were significant differences based on region of residence, mean age, visual acuity, and rate of eyeglass use between the two groups. There was no statistically significant difference in terms of sex or spherical equivalent (diopter) between the two groups (Table 1).

**Table 1:** Comparative analysis between the control and Dry Eye Disease (DED) Groups

	Control	DED	P
Subjects (N, (%))	856 (93.4 %)	60 (6.6 %)	
Region of Urban (%)	67.5	86.7	0.03^a^
Age (years)	9.49 ± 1.00	9.90 ± 0.93	0.03^b^
Sex of Female (%)	46.5	53.3	0.47^a^
Visual Acuity (LogMAR)	0.33 ± 0.46	0.61 ± 0.37	<0.001^b^
SE (diopter)	−2.05 ± 2.15	−2.55 ± 1.84	0.22^b^
Use of Glass (%)	38.1	63.3	0.01^a^
Related with DED Risk Factors			
Use of Smartphones (%)	55.4	96.7	<0.001^a^
Smartphone use/day (hours)	0.62 ± 0.68	3.18 ± 0.97	<0.001^b^
Computer use/day (hours)	0.76 ± 0.34	1.10 ± 0.53	<0.001^b^
TV use/day (hours)	1.07 ± 0.54	1.18 ± 0.40	0.27^b^
Learning/day (hours)	2.31 ± 1.02	3.10 ± 0.50	<0.001^b^
Related with DED Protective Factors			
Sleep/day (hours)	7.88 ± 0.86	7.70 ± 0.61	0.14^b^
Outdoor activity/day (hours)	2.27 ± 1.12	1.47 ± 0.32	<0.001^b^

*DED* dry eye disease, *SE* spherical equivalent, *logMAR* logarithm of the minimum angle of resolution, *TV* television

^a^By Pearson’s Chi-square test, ^b^By independent t test

The rate of smartphone use was 55.4 % in the control group and, 96.7 % in the DED group (*P* < 0.001). Smartphone use time per day was 0.62 ± 0.68 h in the control group and 3.18 ± 0.97 h in the DED group (*P* < 0.001); computer use time per day was 0.76 ± 0.34 h in the control group and 1.10 ± 0.53 h in the DED group (*P* < 0.001). Learning time per day was 2.31 ± 1.02 h in the control group and 3.10 ± 0.50 h in the DED group (*P* < 0.001). Outdoor activity time per day was 2.27 ± 1.12 h in the control group and 1.47 ± 0.32 h in the DED group (*P* < 0.001).

### Comparative analysis between urban and rural groups for pediatric DED

We performed a comparative analysis of DED in 630 school children in urban environments and 286 children in rural environments. Visual acuity (LogMAR) was worse in the urban group, and the rate of eyeglass use was higher in the urban group compared to the rural group (Table 2).

**Table 2:** Comparative analysis of pediatric DED between urban and rural groups

	Urban	Rural	P
Subjects (N)	630	286	
Age (years)	9.51 ± 1.00	9.52 ± 1.00	0.91^b^
Dry eye disease (%)	8.3	2.8	0.03^a^
Visual acuity (logMAR)	0.42 ± 0.57	0.23 ± 0.45	<0.001^b^
SE (diopter)	−2.13 ± 2.34	−1.98 ± 1.58	0.41^b^
Use of glass (%)	42.9	32.9	0.04^a^
Related with DED Risk Factors			
Use of smartphones (%)	61.3	51	0.04^a^
Smartphone use/day (hours)	0.93 ± 1.01	0.47 ± 0.69	<0.001^b^
Computer use/day (hours)	0.84 ± 0.29	0.68 ± 0.46	<0.001^b^
TV use/day (hours)	1.05 ± 0.48	1.15 ± 0.64	0.08^b^
Learning/day (hours)	2.50 ± 0.95	2.05 ± 1.07	<0.001^b^
Related with DED Protective Factors			
Sleep/day (hours)	7.88 ± 0.84	7.84 ± 0.87	0.57^b^
Outdoor activity/day (hours)	2.06 ± 1.13	2.57 ± 0.95	<0.001^b^

*DED* dry eye disease, *SE* spherical equivalent, *logMAR* logarithm of the minimum angle of resolution, *TV* television

^a^By Pearson’s Chi-square test, ^b^By independent t test

The prevalence of DED was 8.3 % in the urban group and 2.8 % in the rural group. This difference was statistically significant (*P* = 0.03). The rate of smartphone use between the two regions also differed significantly, with 61.3 % in urban areas and 51.0 % in rural areas (*P* = 0.04). The mean daily duration of smartphone use was 0.93 ± 1.01 h in the urban group and 0.47 ± 0.69 h in the rural group (*P* < 0.001), while that of computer use was 0.84 ± 0.29 h in the urban group and 0.68 ± 0.46 h in the rural group (*P* < 0.001). The mean daily duration of outdoor activity was 2.06 ± 1.13 h in the urban group and 2.57 ± 0.95 h in the rural group (*P* < 0.001). The average daily duration of learning was 2.50 ± 0.95 h in the urban group and 2.05 ± 1.07 h in the rural group (*P* < 0.001). Age, spherical equivalent, TV watching, and sleeping time did not differ significantly between the two groups (Table 2).

### Comparative analysis of pediatric DED between younger (1st to 3rd) and older (4th to 6th) grade children

We performed comparative analysis of DED in 452 younger grade (1st to 3rd) children and 464 older grade (4th to 6th) children. The visual acuity (LogMAR) was worse in older graders, and the rate of eyeglass use was higher in older graders than in younger graders. The prevalence of DED was 4 % in younger graders and 9.1 % in older graders, which was a statistically significant difference (*P* = 0.03). The rate of smartphone use was 50.9 % in younger graders compared to 65.1 % in older graders. This was also statistically significant (*P* < 0.001) (Table 3).

**Table 3:** Comparative analysis of pediatric DED between younger (1st to 3rd) and older (4th to 6th) grade

	Younger grade	Older grade	P
Subjects (N)	452	464	
Region of Urban (%)	69.0	68.5	0.92^a^
Dry Eye Disease (%)	4	9.1	0.03^a^
Visual Acuity (LogMAR)	0.28 ± 0.42	0.41 ± 0.48	<0.001^b^
SE (diopter)	−1.77 ± 2.12	−2.39 ± 2.11	<0.001^b^
Use of Glass (%)	31	48.3	<0.001^a^
Related with DED Risk Factors			
Use of Smartphones (%)	50.9	65.1	<0.001^a^
Smartphone use/day (hours)	0.57 ± 0.76	1.00 ± 1.06	<0.001^b^
Computer use/day (hours)	0.74 ± 0.37	0.83 ± 0.35	<0.01^b^
TV use/day (hours)	1.03 ± 0.52	1.13 ± 0.55	0.06^b^
Learning/day (hours)	2.08 ± 0.95	2.63 ± 1.00	<0.001^b^
Related with DED Protective Factors			
Sleep/day (hours)	7.93 ± 0.83	7.81 ± 0.87	0.11^b^
Outdoor activity/day (hours)	2.49 ± 1.07	1.94 ± 1.07	<0.001^b^

*DED* dry eye disease, *SE* spherical equivalent, *logMAR* logarithm of the minimum angle of resolution, *TV* television

^a^By Pearson’s Chi-square test, ^b^By independent t test

The mean daily duration of smartphone use was 0.57 ± 0.76 h in younger graders and 1.00 ± 1.06 h in older graders (*P* < 0.001), and the mean daily duration of computer use was 0.74 ± 0.37 h in younger graders and 0.83 ± 0.35 h in older graders (*P* < 0.01). These differences were all statistically significant. The daily duration of learning was 2.08 ± 0.95 h in younger graders and 2.63 ± 1.00 h in older graders (*P* < 0.001). The daily duration of outdoor activity was 2.49 ± 1.07 h in younger graders and 1.94 ± 1.07 h in older graders (*P* < 0.001). Region of residence, mean daily TV watching and sleep duration did not show significant differences between the two groups (Table 3).

### Analysis of risk factors and protective factors for pediatric DED

We performed logistic regression analysis to investigate DED risk factors and protective factors. The mean daily duration of smartphone use was 3.18 ± 0.97 h in the DED group compared with 0.62 ± 0.68 h in the normal group (OR = 13.07, *P* < 0.001) (Table 4).

**Table 4:** Analysis of risk factors and protective factors for pediatric DED

	Normal	DED	OR (95 % CI)	P
Related with DED Risk Factors				
Smartphone use/day (hours)	0.62 ± 0.68	3.18 ± 0.97	13.07 (5.99–28.52)	<0.001^a^
Computer use/day (hours)	0.76 ± 0.34	1.10 ± 0.53	0.94 (0.20–4.42)	0.94^a^
TV use/day (hours)	1.07 ± 0.54	1.18 ± 0.40	0.20 (0.03–1.43)	0.11^a^
Learning/day (hours)	2.31 ± 1.02	3.10 ± 0.50	2.51 (0.90–7.03)	0.08^a^
Related with DED Protective Factors				
Sleep/day (hours)	7.88 ± 0.86	7.70 ± 0.61	0.63 (0.22–1.77)	0.38^a^
Outdoor activity/day (hours)	2.27 ± 1.12	1.47 ± 0.32	0.33 (0.14–0.79)	<0.01^a^

*DED* dry eye disease, *OR* odds ratio, *CI* confidence interval

^a^By Multivariate binominal logistic regression analysis

The mean daily duration of outdoor activity was 1.47 ± 0.32 h in the DED group compared with 2.27 ± 1.12 h in the normal group (OR = 0.33, *P* < 0.01). Mean daily duration of computer use, TV watching, learning and sleep did not relate to DED variably between the two groups (Table 4).

### Comparative analysis before and after cessation of smartphone use over 4 weeks in the DED group

We performed comparative analysis before and after cessation of smartphone use over 4 weeks in the DED group. Punctate epithelial erosion improved after cessation of smartphone use from 93.3 to 0 % (*P* < 0.001). Tear break-up time also improved after cessation from 10.00 ± 3.25 s to 11.33 ± 2.29 s (*P* < 0.001). OSDI score was decreased after smartphone cessation from 30.74 ± 13.36 points to 14.53 ± 2.23 points (*P* < 0.001). Consequently, the rate of DED decreased from 100 to 0 % after smartphone cessation over 4 weeks (*P* < 0.001) (Table 5).

**Table 5:** Comparative analysis before and after cessation of smartphone use for 4 weeks in the DED group

	Control (*N* = 30)	Cessation of smartphone (*N* = 30)	P
PEE (%) baseline	100	93.3	0.50^a^
After 4 weeks	86.7	0	<0.001^a^
TBUT (sec) baseline	9.20 ± 1.93	10.00 ± 3.25	0.42^c^
After 4 weeks	9.47 ± 1.88	11.33 ± 2.29	<0.001^b^
OSDI score baseline	35.76 ± 14.80	30.74 ± 13.36	0.34^c^
After 4 weeks	30.13 ± 13.22	14.53 ± 2.23	<0.001^b^
DED (%) baseline	100	100	
4 weeks	86.7	0	<0.001^a^

*PEE* punctate epithelial erosion, *TBUT* tear break up time, *OSDI* ocular surface disease index, *DED* dry eye disease

^a^By Pearson’s Chi-square test, ^b^By Repeated measure ANOVA, ^c^By Independent t-test

## Discussion

In a previous study, the recent increase in smartphone use caused an increase in reports of symptoms such as irritation, burning sensation, conjunctival injection, decreased visual acuity, strain, and fatigue [11]. To the best of our knowledge, there have been no previous comparative studies of pediatric DED according to region and age. In the Republic of Korea, the internet is well-developed and widely available, so it is common for children to use VDT to view media and internet content. Additionally, the rate of smartphone use has rapidly increased in Korean populations, especially among adolescents and school children.

A reduced blink rate during continuous smartphone use causes faster evaporation of the tear film, which may then lead to DED. In a previous study, we noted video display terminal use and reading reduced the blink rate to 5–6/min (1/3 of the resting state rate) and promoted tear film evaporation and accommodation [12–15]. This may lead to ocular fatigue and myopic shift. Here, we pose the possibility that pediatric smartphone use and reading induces a lower blink rate, which may contribute to or cause pediatric DED. This comparative study of pediatric DED associated with smartphone use evaluates inter-region and inter-age variables. Logistic regression analysis was performed to investigate the risk and protective factors associated with pediatric DED.

The rate of smartphone use was significantly higher in the DED group than the control group. Continuous smartphone use is thought to decrease the blink rate and promote evaporation of the tear film, thus inducing DED. There was a significant difference in the mean duration of outdoor activity and learning between the two groups; however, on logistic regression analysis, the mean duration of smartphone use (OR = 13.07) was the only strong risk factor for pediatric DED. The mean duration of computer use, TV watching and learning was not significantly different on multivariate logistic regression analysis. In addition, increased outdoor activity time reduced the rate of pediatric DED (OR = 0.33).

A greater proportion of students in the DED group were urban versus rural residents. We suggest that that the difference in smartphone use between regions led to the difference in DED prevalence. The rate of eyeglass use was higher in urban students and visual acuity (LogMAR) was better in the rural group. The rate of DED prevalence was higher in the urban group compared to the rural group, and the mean daily duration of smartphone use, computer use, and learning was significantly longer in urban populations. Outdoor activity time was longer in the rural group. The longer duration of smartphone use, computer use and learning relative to low outdoor activity may have increased the prevalence of DED and the rate of eyeglass use in urban populations.

DED prevalence was also found to be higher in females than males. We surveyed our study population to identify these differences in Korean children. In our study, DED prevalence in children was higher in females; however, there was no statistical significance. There was no difference between the two groups with regard to VDT use. In general, DED prevalence was higher in female adults compared to male adults.

The mean age was higher in the DED group than in the normal group, and DED prevalence was higher in older graders than younger graders. The rate of smartphone use was higher and the mean daily duration of VDT was longer in older grades, which may have led to the higher DED prevalence in older graders.

Smartphones are used with short watching distances due to their small LED screens, thus inducing ocular fatigue, glare, and irritation. VDT is associated with an increased incidence of DED and decreased visual acuity. Gur [16] et al. reported that VDTs require reduced accommodation and fusional convergence and induce a −0.12 diopter myopic shift.

A limitation of our study is that only one school in each region was surveyed. If we surveyed a greater number of schools in urban or rural areas, our data would be more representative. Our study was aimed at school children, so there may have been difference in self-reporting and expression of ocular discomfort and comprehension of the questionnaire between younger graders and older graders, which could have affected our estimates of DED prevalence. We did not consider a myriad of other factors, including environmental and socioeconomic status. We have shown an association and that in those who have dry eyes we have shown that cessation of smartphone use improves signs and symptoms; however, there are many other variables that we have not assessed. In a smartphone cessation study, it would be better to use controls who had subjective complaints or higher OSDI scores and no obvious disease on clinical exams to rule out accommodative fatigue or placebo effect.

We used subjective symptom criteria (modified OSDI score) and objective signs (low TBUT or PEE) to diagnose DED. These criteria were based on adults and subjective signs of DED were not obvious. Thus, the true prevalence of DED in school children may be increased or decreased due to the use of adult-targeted diagnostic criteria. In our previous study, we confirmed that DED coexists with allergic conjunctivitis [17]; however, because DED and ocular allergies have similar symptoms, it was difficult to separate children with DED from those with ocular allergies [18, 19]. Thus, the prevalence of DED may have been affected by the prevalence of allergies in our study population. We did not consider confounding factors such as socioeconomic status, family composition, dual income family status, or concurrent artificial tear use.

## Conclusion

In conclusion, increased VDT use such as smartphones or computers in Korean children was found to be associated with the occurrence of ocular surface symptoms. Increased use of smartphones is a serious issue that may result in ocular problems: the prevalence of DED was 6.6 %. The mean duration of VDT use per day, especially smartphone use, was greater in the DED group than in the normal group. Longer daily smartphone use may be a risk factor for DED. The prevalence of DED was higher in the urban group and in older children. Older-grade children in the urban group used smartphones for longer time periods than younger-grade children in rural areas. DED prevalence was higher in older grade children from urban groups compared to younger grade children in rural groups. After cessation of smartphone use for 4 weeks in DED patients, both subjective symptoms and objective signs improved.

Therefore, close observation and caution during VDT use, especially smartphones, is recommended for older children in urban areas. DED in children must be detected early and should be treated with appropriate medical and environmental interventions and education.

## Additional file

Additional file 1: OSDI, questionnaire and protocol. This file included OSDI index, ophthalmologic questionnaire and study protocol. (DOCX 83 kb)

## Abbreviations

CI: Confidence interval
DED: Dry eye disease
logMAR: logarithm of the minimum angle of resolution
OR: Odds ratio
OSDI: Ocular surface disease index
SE: Spherical equivalent
TBUT: Tear break up time
TV: Television
VDT: Video display terminal
