# Supplementary material for: Smartphone use is a risk factor for pediatric dry eye disease according to region and age: a case control study
Source: BMC Ophthalmol. 2016 Oct 28;16:188. doi: 10.1186/s12886-016-0364-4 (PMC5084437; doi:10.1186/s12886-016-0364-4)
Supplement: Additional file 1: — OSDI, questionnaire and protocol. This file included OSDI index, ophthalmologic questionnaire and study protocol. (DOCX 83 kb) [file 12886_2016_364_MOESM1_ESM.docx]

**Ocular Surface Disease Index, Questionnaire and Protocol**

**Name: attach autokeratometry Sex/Age:**

**Best Corrected Visual Acuity (BCVA)**

Rt. :

Lt. :

**Slit Lamp Examination**

Lid : cilio-corneal touch (-/-)

Conjunctiva : papilla (-/-) follicle (-/-) injection (-/-)

Cornea : erosion (-/-)

Lens : (-/-)


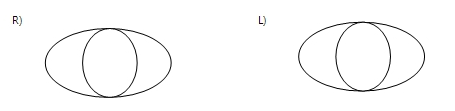


**Tear Break Up Time (TBUT)**

Rt. : sec

Lt. : sec


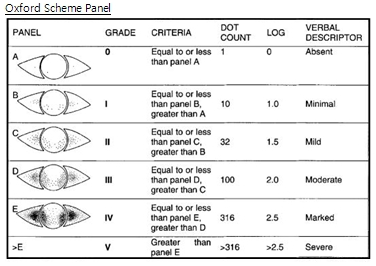


**Punctate Epithelial Erosion (PEE) Grade**

Rt. :

Lt. :

**Strabismus: ortho/ XT/ X/ ET/ E**

**Ocular Surface Disease Index (OSDI)**

Have you experienced any of the following during the last week?

|  | All of the time | Most of the time | Half of the time | Some of the time | None of the time |
| --- | --- | --- | --- | --- | --- |
| Eyes that are sensitive to light? | 4 | 3 | 2 | 1 | 0 |
| Eyes that feel gritty? | 4 | 3 | 2 | 1 | 0 |
| Painful or sore eyes? | 4 | 3 | 2 | 1 | 0 |
| Blurred vision? | 4 | 3 | 2 | 1 | 0 |
| Poor vision? | 4 | 3 | 2 | 1 | 0 |
| Dryness? | 4 | 3 | 2 | 1 | 0 |
| Tearing? | 4 | 3 | 2 | 1 | 0 |
| Red eye or injection? | 4 | 3 | 2 | 1 | 0 |
| Fatigue or headache? | 4 | 3 | 2 | 1 | 0 |

Subtotal score for answers = (A)

Have problems with your eyes limited you in performing any of the following during the last week?

|  | All of the time | Most of the time | Half of the time | Some of the time | None of the time | N/A |
| --- | --- | --- | --- | --- | --- | --- |
| Reading? | 4 | 3 | 2 | 1 | 0 | N/A |
| Use smartphone? | 4 | 3 | 2 | 1 | 0 | N/A |
| Use computer? | 4 | 3 | 2 | 1 | 0 | N/A |
| Watching television? | 4 | 3 | 2 | 1 | 0 | N/A |

Subtotal score for answers = (B)

Have your eyes felt uncomfortable in any of the following situations during the last week?

|  | All of the time | Most of the time | Half of the time | Some of the time | None of the time | N/A |
| --- | --- | --- | --- | --- | --- | --- |
| Windy condition? | 4 | 3 | 2 | 1 | 0 | N/A |
| Areas with low humidity? | 4 | 3 | 2 | 1 | 0 | N/A |
| Areas that are air conditioned? | 4 | 3 | 2 | 1 | 0 | N/A |

Subtotal score for answers = (C)

Add subtotals A, B, and C to obtain D (D = sum of scores for all questions answered) = (D)

**OSDI Score = (D) x 100 / (# of question answered) x 4 = ( )**

**Ophthalmologic Questionnaire**

Please put in a tick in the box √ next to the answer of your choice.

1. Medical treatment in ophthalmologic clinic?

Yes □

No □

1. Diagnosis?

Strabismus □

Amblyopia □

Refractive error □

Allergic conjunctivitis □

Viral conjunctivitis □

Trichiasis □

Etc □ 🡪

N/A □

1. Use glass?

Yes □ 🡪 during ( ) years

No □

1. Lagophthalmos?

Yes □

No □

1. Have atopic disease?

Yes □

No □

1. Have allergic disease (rhinitis, conjunctivitis, dermatitis)?

Yes □

No □

1. Received medical treatment for allergic disease?

Yes □

No □

1. Use ophthalmic solution for allergic conjunctivitis?

Yes □

No □

1. Itching sensation?

All of the time □

Most of the time □

Half of the time □

Some of the time □

None of the time □

1. Use smartphone?

Yes □ 🡪 go No. 11

No □ 🡪 go No. 12

1. Mean daily duration of smartphone use?

0-1 hours □

1-2 hours □

2-3 hours □

3- hours □

1. Mean daily duration of personal computer use?

0-1 hours □

1-2 hours □

2-3 hours □

3- hours □

1. Mean daily duration of watching television?

0-1 hours □

1-2 hours □

2-3 hours □

3- hours □

1. Mean daily duration of sleeping?

0-6 hours □

6-7 hours □

7-8 hours □

8-9 hours □

9- hours □

1. Mean daily duration of outdoor activitiy?

0-1 hours □

1-2 hours □

2-3 hours □

3- hours □

1. Mean daily duration of learning?

0-1 hours □

1-2 hours □

2-3 hours □

3- hours □
